# Effectiveness of a pharmacist-led tele-psychiatric clinic in managing drug-related problems

**DOI:** 10.1080/20523211.2025.2460038

**Published:** 2025-02-07

**Authors:** Majed Al Shakhori, Savera Arain, Suhaj Abdulsalim, Mohammed Salim Karattuthodi, Marwa Al Dhamen, Shoruq Almutairi, Shabeer Ali Thorakkattil, Dina Alahmdi, Vijayanarayana Kunhikatta, Abdul Sammad AlJishi

**Affiliations:** a Pharmacy Services Department, Johns Hopkins Aramco Healthcare (JHAH), Dhahran, Saudi Arabia; b Department of Pharmacy Practice, College of Pharmacy, Qassim University, Buraydah, Saudi Arabia; c Department of Pharmacy Practice, Manipal College of Pharmaceutical Sciences, Manipal Academy of Higher Education, Manipal, Karnataka, India; d Pharmacy Services Department, King Fahad Specialist Hospital, Dammam, Saudi Arabia; e Department of Pharmacy Practice and Clinical Pharmacy, Faculty of Pharmacy, Universiti Teknologi MARA (UiTM), Selangor, Malaysia; f Psychiatry Department, Johns Hopkins Aramco Healthcare (JHAH), Dhahran, Saudi Arabia

**Keywords:** Drug-related problems, mental health, pharmaceutical services, clinical pharmacy, psychiatry, pharmacist interventions, Krska-classification system, retrospective study

## Abstract

**Background:**

The study aimed to evaluate the effectiveness of telepsychiatry combined with the expertise of psychiatric clinical pharmacists in identifying and addressing drug-related problems (DRPs) associated with psychotropic medications. Additionally, the research assessed physicians' acceptance of pharmacists' recommendations for managing these DRPs.

**Methods:**

A cohort retrospective study was conducted at a leading tertiary care hospital in Saudi Arabia spanning from January 2023 to January 2024 in a psychiatry setting. The study comprehensively examined all instances of interventions for DRPs facilitated through patient-initiated telepsychiatry encounters with psychiatric clinical pharmacists. Detailed and meticulously recorded notes from patient chart reviews, documented by the pharmacist in the Electronic Health Record (EHR), during each encounter, were reviewed. These notes provided significant information on psychiatric diagnosis, identified DRPs and the specific interventions and recommendations proposed by the clinical pharmacist to the attending physician. The Krska classification was utilised to classify and analyse the identified DRPs, ensuring a structured and systematic approach to the study's findings.

**Results:**

A total of 259 pharmacist interventions were made, and the results revealed a remarkably high acceptance rate of 98.5% among physicians. The most common intervention (16.21%) involved targeted education to improve medication adherence. Additionally, substantial efforts were directed towards rectifying inappropriate dosage regimens, accounting for 13.51% of DRPs resolved by the pharmacist. Noteworthy interventions also encompassed the identification and management of potential or suspected adverse reactions, comprising 12.35% of the interventions, along with interventions addressing concerns regarding potentially ineffective therapy, which constituted 11.59%.

**Conclusion:**

The study underscores the critical role of pharmacists in psychiatric care, with high physician acceptance of their interventions. The diverse range of DRPs highlights the need to expand clinical pharmacy services and integrate pharmacists into psychiatric teams. Our findings clearly demonstrate that integrating pharmacists into psychiatric care settings is beneficial. This approach enhances DRP identification and management, ultimately enhancing patient care and treatment outcomes.

## Background

Mental health disorders pose a significant global challenge, with the majority of people unable to access even basic treatment due to insufficient or nonexistent mental health care systems. It impacts approximately 450 million people worldwide, representing 13% of the global disease burden, and is projected to escalate to 15% by 2030 (Carbonell et al., 2020; Hui et al., 2024; Rubio-Valera et al., 2014). Mental health disorders often lead to additional health complications, increased costs, and higher healthcare utilisation (Kelly & Love, 2019; Werremeyer et al., 2020). Additionally, pharmacists have noted a higher occurrence of drug-related problems (DRPs) among individuals undergoing treatment for mental illnesses (Bell et al., 2006; Jayakumar et al., 2021). DRP is defined as the occasion or situation involving drug treatment that really or conceivably obstructs health care outcomes (Dagnew et al., 2022). It also defined as ‘An event or circumstance involving drug therapy that actually or potentially interferes with desired health outcomes’ (Al-Worafi, 2020). DRPs can cause significant patient morbidity or mortality in addition to increased healthcare expenditures (Ruths et al., 2007; Westerlund, 2019). The higher occurrence of DRPs in psychiatric patients can be attributed to the pharmacokinetic and pharmacodynamics characteristics of psychiatric medications or from the utilisation of the same classes of psychotropic medications in the treatment of different psychiatric disorders (Dagnew et al., 2022; Jayakumar et al., 2021). Despite the rising number of individuals diagnosed with mental disorders, there is a shortage of mental healthcare providers and services, resulting in what is known as the ‘mental health gap’ (McGrane & Mertens, 2018). The shortage of mental healthcare providers highlights the crucial need for collaboration among existing healthcare professionals and mental health providers to fill this gap (Caley et al., 2010).

In recent decades, the role of psychiatric pharmacists has expanded into various specialised practice areas. These activities include managing clozapine clinics, monitoring long-acting injectable medication treatment plans, prescribing independently, providing chronic disease management in various outpatient settings through patient encounters, conducting one-on-one consultations, offering medication review services, delivering mental health support to students on educational campuses, educating other healthcare team members, and addressing potential DRPs through pharmaceutical care interventions (El-Den et al., 2021; Hui et al., 2024; Werremeyer et al., 2020).

Pharmacists remain a crucial component of mental health collaborative teams by offering their expertise to address the potential adverse effects and complications linked to psychiatric medications. Their primary responsibility includes providing pharmaceutical care and involving the responsible provision of drug therapy to achieve specific patient outcomes and improve overall quality of life. Previous studies have already reported the role of clinical pharmacists in improving patient outcomes by preventing potential DRPs and managing actual DRPs and thereby improving quality of life of individual patients in many mental diseases (Adler et al., 2004; Alshahrani et al., 2019; Vitija et al., 2022). Furthermore, their responsibilities, as supported by extensive research, encompass ensuring the appropriateness, effectiveness, and safety of medications for individual patients as well as identifying, resolving, and preventing various DRPs that pose a significant burden on the healthcare system (Kamusheva et al., 2020; Langarizadeh et al., 2017; Liu et al., 2021). A study by Werremeyer et al., demonstrated significant improvements in patient outcomes by integrating psychiatric pharmacist interventions with the inter-professional healthcare teams (Werremeyer et al., 2020). Furthermore, a meta-analysis of pharmaceutical care interventions demonstrated a positive impact on the mental health domain of the SF-36 quality-of-life instrument (El-Den et al., 2021). Given the significant impact of pharmacist interventions in psychiatric settings, this study was conducted to investigate the specific types of interventions pharmacists made to address DRPs through pharmacist-led tele-psychiatric consultation, evaluate the acceptance rate of these interventions by physicians, and provide valuable insights into the significant contributions of pharmacists in psychiatric care.

## Methods

### Study setting and design

A retrospective study was conducted at the Johns Hopkins Aramco Healthcare (JHAH), a tertiary care hospital in Saudi Arabia, to assess pharmacist interventions for DRPs in patients with psychiatric disorders. At JHAH, the board-certified psychiatric pharmacist provides psychopharmacology consultations, serving as an integral member of the inter-professional healthcare team. Patients may be referred by their provider or directly request an appointment with the psychiatric pharmacist through a telephone encounter. Telephone encounter is a component of the pharmacist-led telepsychiatry clinic, which offers a direct phone line with a designated number and an interactive voice response (IVR) system to direct patient phone calls to the psychiatric clinic where the pharmacist receives patients’ calls during work hours (Arain et al., 2022). By enabling patients to access the telephone encounter system, including tele-psychiatric consultation services which were the primary focus of this study. JHAH has introduced an innovative approach to address patients’ DRPs.

By utilising telephonic consultations, the pharmacist can comprehensively review the patient's medical history, recent vital signs, and laboratory results while addressing drug information queries, providing patient counselling, and resolving DRPs. During each encounter, the psychiatric pharmacist thoroughly evaluates medication use by performing the root cause analysis of the DRP. Once the evaluation is complete, the pharmacist would promptly notify the treating physician about the DRP and suggest an intervention. The interventions were made by the board-certified pharmacist after conducting a medication review based on a three-step process (Soerensen et al., 2016). By utilising this process, the pharmacist was able to make informed decisions on the appropriate course of action for the intervention. The proposed intervention was either approved or rejected. If approved, the pharmacist proceeded with the appropriate intervention and recommendation to effectively resolve the DRP. Additionally, psychiatric rating scales are administered as deemed necessary based on the purpose of the consultation. Each patient encounter was meticulously documented in the electronic health record (EHR), with recommendations or modifications recorded using the electronic clinical intervention system seamlessly integrated into the Epic EHR platform.

### Data collection and analysis

Patients were identified through a detailed retrospective review of telepsychiatry visits to a psychiatric pharmacist clinic spanning from January 2023 until January 2024. Our study focused on the interventions documented in the EHR by psychiatric pharmacist, specifically where interventions were made to address drug-related problems (DRPs). The data collection process included the extraction of key demographic data, including gender and age, from patient charts within the JHAH EHR system by study investigators. Following this, detailed patient notes from each patient encounter, documented by the psychiatric pharmacist during each consultation, were thoroughly reviewed. The review's objective was to extract specific details such as the patient’s psychiatric diagnoses, the number of chronic medications being taken, the specific DRPs identified, and the description of the pharmacist’s interventions and recommendations. The documentation of the pharmacist’s interventions, including the physician’s acceptance or rejection, facilitated the assessment of the acceptance rates during the retrospective review. The DRPs were classified using the criteria set by Krska et al., enabling a standardised framework for categorising the DRPs for the study (Krska et al., 2002). When compared to other classification systems, the Krska method was the most thorough in covering the DRPs that arose in this particular patient setting, hence it was used to categorise the DRPs that the pharmacist handled. Additionally, the Krska classification closely aligns with this study as it is rooted in drug-use evaluation managed by the pharmacist. The primary investigator supervised the data collection process to maintain accuracy and reliability. By using this approach, a comprehensive analysis was conducted to analyse the role of psychiatric pharmacist in addressing the DRPs identified through telepsychiatry.

### Inclusion and exclusion criteria

The study analysis included the evaluation of the interventions performed by psychiatric clinical pharmacist for DRPs in mental health patients using the telephone encounter feature in the Epic EHR system at JHAH. Any interventions that were incomplete or lacked proper documentation were excluded.

### Statistical analysis

Statistical Package for Social Services (SPSS) version 20 were used for data entry, curation, and analysis. The study variables were presented by descriptive statistics such as mean and standard deviation. The categorical data were presented as frequencies and percentages. Further, a chi-square test was employed to find the association between two categorical variables, and a *p*-value of less than 0.01 was considered statistically significant.

## Results

### Distribution of demographic details of the patients

There were 259 total tele-pharmacy consultations that were recorded as an intervention for a DRP by the psychiatric pharmacist during the study period. The mean age of patients was 44.42 ± 19.35 years, with the male population (*n* = 149, 57.5%) being predominant. The mean body weight and body mass index of the study population was 78.71 ± 21.70 kg and 30.11 ± 17.19 kg/m^2^.

### Distribution of psychiatric patients based on their diagnosis

**Figure 1.**
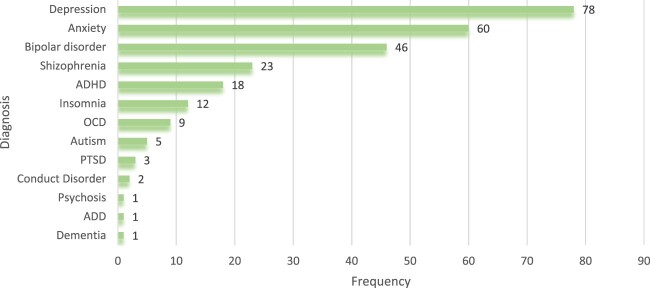
Diagnosis of psychiatric patients enrolled in the study.

It was noted that there was a high prevalence of depression (*n* = 78, 29.4%) and anxiety (*n* = 60, 22.6%). Additionally, there were a few patients with psychosis, dementia, and inattentive-type attention deficit disorder. The majority had a single psychiatric condition. The details of the diagnosis are given in Figure 1.

### Distribution of psychiatric patients by prescribed medications

In the current study, pharmacists made an intervention for 259 DRPs. Among these, 77.2% (*n* = 200) of patients were using a single antipsychotic medication, while 22.8% (*n* = 59) were on a combination of more than one psychiatric medication. Within this cohort of patients on a single psychiatric medication, second-generation antipsychotic agents and antidepressants were found to be the most frequently prescribed medications. Predominantly, the second-generation antipsychotic quetiapine (*n* = 25, 9.7%) was the primary medication, followed by antidepressants escitalopram (*n* = 24, 9.3%) and fluoxetine (*n* = 16, 6.2%). Notably, among the various psychotropic medications, lorazepam (*n* = 14, 5.4%) was the prevailing antianxiety medication, with zolpidem (*n* = 9, 3.5%) most prescribed for insomnia management (Figure 1). Among patients receiving a combination of psychotropic medications, the most common prescription included second-generation antipsychotic olanzapine (*n* = 10, 3.9%) and quetiapine (*n* = 9, 3.5%). Moreover, zolpidem (*n* = 4, 1.5%) was also noted as a frequently utilised medication among patients on combination psychiatric regimens (Table 1).

**Table 1.** Frequency and percentage distribution of patients on their first or second psychiatric medications.

Psychiatric medication	First		Second	
	Frequency (*n*)	Percent	Frequency (*n*)	Percent
Atomoxetine	3	1.2	–	–
Clonidine	1	0.4	–	–
Methylphenidate	7	2.7	–	–
Lisdexamfetamine	8	3.1	1	0.4
Rivastigmine	1	0.4	–	–
Benztropine	1	0.4	1	0.4
Escitalopram	24	9.3	3	1.2
Venlafaxine	9	3.5	2	0.8
Fluoxetine	16	6.2	3	1.2
Paroxetine	10	3.9	1	0.4
Desvenlafaxine	9	3.5	2	0.8
Sertraline	10	3.9	2	0.8
Trazadone	5	1.9	–	–
Duloxetine	1	0.4	1	0.4
Amitriptyline	4	1.5	1	0.4
Bupropion	4	1.5	1	0.4
Mirtazapine	6	2.3	2	0.8
Vortioxetine	2	0.8	1	0.4
Hydroxyzine	1	0.4	–	–
Zolpidem	9	3.5	4	1.5
Lorazepam	14	5.4	3	1.2
Clonazepam	13	5	1	0.4
Eszopiclone	1	0.4	–	–
Diazepam	1	0.4	–	–
Bromazepam	1	0.4	–	–
Temazepam	2	0.8	1	0.4
Haloperidol	4	1.5	–	–
Pregabalin	5	1.9	–	–
Carbamazepine	1	0.4	–	–
Lithium	7	2.7	1	0.4
Lamotrigine	4	1.5	3	1.2
Valproate	3	1.2	1	0.4
Clozapine	4	1.5	–	–
Paliperidone	13	5	1	0.4
Risperidone	6	2.3	–	–
Aripiprazole	14	5.4	–	–
Quetiapine	25	9.7	9	3.5
Olanzapine	8	3.1	10	3.9
Amisulpiride	1	0.4	–	–
Melatonin	1	0.4	2	0.8
Lurasidone	–	–	1	0.4
Gabapentin	–	–	1	0.4
Total	**259**	**100**	**59**	**23**.**3**

### Distribution of DRPs according to the Krska system of classification

**Figure 2.**
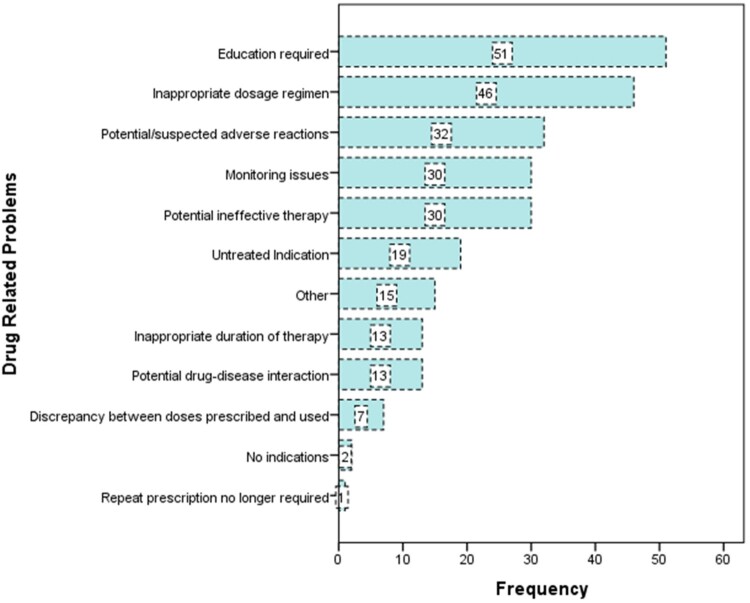
Drug related problems identified as per KRSKA classification.

A total of 259 DRPs requiring pharmacist intervention were identified during the study period. The most common DRP among patients on psychotropic medication was the need for additional education (*n* = 51, 16.21%), particularly to address medication adherence issues. This was followed by inappropriate dosage regimens (*n* = 46, 13.51%). Other significant DRPs included issues related to potential or suspected adverse reactions (*n* = 32, 12.35%), potentially ineffective therapy (*n* = 30, 11.59%), and the need for effective medication monitoring (*n* = 30, 11.59%). These DRPs collectively made up a major component of DRPs requiring pharmacist intervention. Detailed information on the types of DRPs is provided in Figure 2.

### Psychiatric drug classes and DRPs identified

The majority (*n* = 100, 38.6%) of identified DRPs involved the use of antidepressant drugs, followed by second-generation antipsychotic drugs (*n* = 71, 287.4%). Whereas the requirement of education and inappropriate dosage regimen were the prominent DRPs, contributing to 51 (19.7%) and 46 cases (17.8%). Details are given in Table 2.

**Table 2.** Psychiatric drug classes and its associated DRPs identified in the study.

Medication/DRPs	D	A	R	U	E	IDO	DU	M	N	O	PD	PI	Total (*n*)	Percent
Antidepressant	4	12	1	10	19	14	7	13	0	5	5	10	100	38.6
second generation antipsychotic	0	11	0	2	13	19	2	8	0	5	2	9	71	27.4
Benzodiazepine	3	0	0	2	6	5	0	5	1	1	3	5	31	12.0
ADHD nonstimulant	0	4	0	0	5	2	2	3	0	2	1	0	19	7.3
ADHD stimulant	0	0	0	1	5	3	1	0	1	0	0	4	15	5.8
non-benzodiazepine hypnotics	0	4	0	2	1	1	0	0	0	0	0	2	10	3.9
GABA Analog	0	0	0	1	1	1	1	0	0	0	1	0	5	1.9
First generation antipsychotic	0	1	0	1	0	0	0	1	0	1	0	0	4	1.5
Acetylcholinesterase Inhibitor (Central)	0	0	0	0	0	1	0	0	0	0	0	0	1	0.4
Anti-cholinergic	0	0	0	0	0	0	0	0	0	1	0	0	1	0.4
Sleep aid	0	0	0	0	1	0	0	0	0	0	0	0	1	0.4
Anxiolytic	0	0	0	0	0	0	0	0	0	0	1	0	1	0.4
Total (n)	7	32	1	19	51	46	13	30	2	15	13	30	259	100
Percent	2.7	12.4	0.4	7.3	19.7	17.8	5.0	11.6	0.8	5.8	5.0	11.6		100

D, Discrepancy between doses prescribed and used; A, Potential/suspected adverse reactions; R, Repeat prescription no longer required; U, Untreated Indication; E, Education required; IDo, Inappropriate dosage regimen; Du, Inappropriate duration of therapy; M, Monitoring issues; N, No indications; O, Other; PD, Potential drug-disease interaction; PI, Potential ineffective therapy.

### Physicians’ acceptance rate of pharmacists’ interventions

It was identified that 98.45% of the pharmacist’s interventions was accepted by the physician.

## Discussion

DRPs represent significant clinical concerns in the pharmaceutical care process. Identifying, resolving, and preventing DRPs are vital to improve patient outcomes. Previous studies have extensively recognised the role of pharmacists in identifying and resolving DRPs (Hailu et al., 2020; Jimmy et al., 2023; Zhang et al., 2023). Pharmacist-led clinical interventions are a significant component of advanced pharmacist services, aiming to optimise patient care in a clinical setting. These interventions greatly improve outcomes and quality of life for psychiatric patients. To the best of our knowledge, this is the first study from Saudi Arabia that unequivocally confirms the role of pharmacist’s intervention in managing DRPs and improving outcomes in psychiatric patients through tele-psychiatric consultation.

Most of our study population were middle-aged (44.42 ± 19.35) males. This was similar to previous studies conducted by Jayakumar et al. and Mukherjee et al., where the researchers found the age group between 30 and 39 for psychiatric patients (Jayakumar et al., 2021; Mukherjee et al., 2015). Whereas another study reported from India found that most of their patients belonged to the age group of 40–60 (Mateti et al., 2015). In contrast, a study by Wali et al. reported that most of their patient population were 20–30 years old (Wali et al., 2018). The variation may be attributed to the difference in predisposing factors and geographic differences in the pattern of psychiatric disorders. Moreover, males were dominant (57.5%) in our study. This was similar in previous studies by Jayakumar et al. (73.2%) and Wali et al. (60%) (Jayakumar et al., 2021; Wali et al., 2018). Our study found depression (29.4%) as the major psychiatric disorder, followed by anxiety (22.6%). Whereas a study by Khoda et al. reported schizophrenia and bipolar disorders as the most prevalent psychiatric disorders (Khoda et al., 2014). Sigh et al. found alcohol-dependent disorders and schizophrenia as the most prevalent psychiatric disorders (Singh et al., 2019). The difference could be due to the fact that in Saudi Arabia, the use of alcohol and drug abuse is punishable under the law when compared to other countries. The pattern of psychiatric disorders differs due to many predisposing factors and regional differences.

Most of our study population were using single antipsychotics (77.2%) when compared to multiple antipsychotics (22.8%). Second-generation antipsychotics (Quetiapine & escitalopram) (9.7%) were the most prescribed psychiatric medications in our population, followed by antidepressants (9.3%). At the same time, another study from India by Jayakumar et al. reported that atypical antipsychotics were the most (35.7%), and Wali et al. reported that typical antipsychotics topped the list in the prescription analysis (Jayakumar et al., 2021; Wali et al., 2018). Among the drug classes with DRPs, antidepressants were the most (38.6%) in our study, followed by second-generation antipsychotics (27.4%). Among the prescribed psychotropic medications, quetiapine and escitalopram were the most commonly prescribed, followed by fluoxetine in our study. This can be correlated to the fact that quetiapine, due to its safer and good tolerability profile, has become a preferred choice for the treatment of psychiatric disorders, including schizophrenia and bipolar disorder (Divac et al., 2014; Muneer, 2015). The most common DRPs for pharmacist intervention involving quetiapine were dose adjustments for insomnia or lack of response (mainly for psychosis), adverse effects like dry mouth, and providing necessary education.

Depression is not only one of the most common mental disorders and a debilitating disease, but it has also become the second-highest cause of disability worldwide. It is predicted to be the leading cause of morbidity by 2030 (Marasine et al., 2021; Yin et al., 2023). Various studies showed significant improvement in depression response and remission at six months in patients who received care from a collaborative team, including a pharmacist, compared to those who received usual care (Fortney et al., 2007, 2013; Kanwal et al., 2016; Pyne et al., 2011). Moreover, a retrospective study analysing the impact of psychiatric pharmacist intervention at an academic multispecialty outpatient clinic demonstrated substantial improvement in patient outcomes, with a reduction in depression and anxiety scores following consultations with psychiatric pharmacists (Hui et al., 2024). Similarly, our study found that depression was the most prevalent diagnosis among psychiatric patients. Furthermore, escitalopram had the highest number of pharmacist consultations, owing to its frequent prescribing. A scoping review by Marasine et al analysed antidepressant prescribing patterns across different countries. The analysis found that selective serotonin reuptake inhibitors (SSRIs) are the most dominant antidepressants prescribed compared to tricyclic antidepressants (TCAs), selective nortriptyline reuptake inhibitors (SNRIs), and other atypical antidepressants because they offer several advantages, particularly fewer associated adverse effects (Marasine et al., 2021). While rare, QTc prolongation and serotonin syndrome are among the potentially severe adverse effects of SSRIs (Landy et al., 2024). An important finding from this study (Table 3) involves a pharmacist intervention when noting prolonged QTc intervals while reviewing patients’ charts. The pharmacist subsequently switched the medication from escitalopram to sertraline after obtaining the physician’s consent. Sertraline has shown minimal clinically significant QTc prolongation in most studies at standard doses [36, 37].

**Table 3.** Examples of clinical pharmacist interventions performed in the study.

Case.no.	Clinical concern: seeking guidance on treatment matters	Diagnoses	Drug-related problem	Clinical Pharmacist intervention	Final acceptance (yes/no)
1	ACE Inhibitors are contraindicated in patients on lithium. Patient was on both medications concomitantly.	Bipolar disorder	Patient was on lithium 900 mg at bedtime and started on enalapril 5 mg, further adjusted to 20 mg per day.	Discontinued ACE inhibitor and advised to switch to amlodipine or nifedipine.	Yes
2	Lamotrigine can cause serious rashes, including hospitalisation and discontinuation of treatment. This can include Stevens-Johnson syndrome (SJS).	Bipolar disorder	Patient was started on lamotrigine 50 mg bid. Patient developed SJS.	Discontinued lamotrigine and advised the patient to go to the emergency room immediately.	Yes
3	Paliperidone long-acting injection should be discontinued for patients with a creatinine clearance (CrCl) of less than 50 ml/min.	Schizophrenia, substance abuse	Patient was on Paliperidone long-acting injection of 100 mg monthly. However, the patient became renally impaired, with CrCl dropping to below 50 ml/min.	Held paliperidone and switched to oral antipsychotic risperidone.	Yes
4	High doses of antidepressants, especially in individuals with bipolar disorder, can potentially induce manic or hypomanic episodes. This phenomenon is known as ‘antidepressant-induced mania’ or ‘antidepressant-induced mood switching.’	Bipolar depression	Patient was initially started on venlafaxine 225 mg.	Held venlafaxine and started antipsychotic to treat bipolar depression (Lurasidone)	Yes
5	Olanzapine, an atypical antipsychotic medication, is generally not recommended for use in patients with delirium, including those with Alzheimer's dementia.	Delirium, agitation and Alzheimer’s dementia	Treatment plan included consideration of starting olanzapine 5 mg intramuscular injection.	Changed to haloperidol 2.5–5 mg intramuscular with baseline EKG.	Yes
6	The development of high prolactin levels (hyperprolactinemia) in a patient on a long-acting injection of paliperidone is a known side effect of antipsychotic medications, including paliperidone. Hyperprolactinemia can have various effects, and one common manifestation in females is menstrual irregularities, including the cessation of periods.	Schizophrenia	Patient undergoing treatment with the long-acting injection of Paliperidone experienced an elevation in prolactin levels, leading to the cessation of her menstrual periods.	Switched to aripiprazole long-acting injection	Yes
7	Clozapine, an atypical antipsychotic, is known to have the potential to induce neutropenia, a condition characterised by a low count of neutrophils.	Schizophrenia	Patient developed moderate neutropenia with clozapine (1200 cell/m^3^)	Increased ANC monitoring to three times per week until > 1500 cell/m^3^	No
8	The patient has schizophrenia and is currently on polypharmacy, which means they are taking three or more antipsychotic medications. Despite this, they are not responding well to the antipsychotic treatment.	Schizophrenia	The patient was taking three different antipsychotic medications: olanzapine 20 mg, haloperidol 10 mg twice daily, and risperidone long-acting injection 25 mg every two weeks.	Switched from risperidone long-acting injection and haloperidol tablets to depot injection of haloperidol and continued with olanzapine oral tablets.	Yes
9	The patient was experiencing chronic insomnia and depression and is currently on multiple medications simultaneously.	Insomnia, Depression	Patient with chronic insomnia and depression on zolpidem 10 mg and temazepam 30 mg.	Discontinued the use of temazepam and initiated the antidepressant mirtazapine at a dose of 15 mg.	No
10	Taking more than one antidepressant from the same group simultaneously is generally not recommended due to the increased risk of adverse effects and potential interactions. Combining multiple antidepressants from the same class, such as selective serotonin reuptake inhibitors (SSRIs) or serotonin-norepinephrine reuptake inhibitors (SNRIs), can lead to a phenomenon known as ‘serotonin syndrome.’	Obsessive-compulsive disorder and depression	Patient was on 3 antidepressants: fluvoxamine, sertraline, and venlafaxine simultaneously.	Discussed with the physician to gradually discontinue two of the antidepressants. The treating physician agreed to discontinue one of the antidepressants and continued the patient on the remaining two antidepressants until the next evaluation visit.	Yes
11	Concurrently using risperidone and clozapine poses a risk of cardiac side effects.	Schizophrenia	The patient, diagnosed with treatment-resistant schizophrenia, did not exhibit a response to high-dose olanzapine. Subsequently, risperidone was initiated and titrated up to 6 mg, yet there was no improvement. As a result, clozapine was introduced as an alternative treatment option.	Initially, discontinued risperidone and closely monitored the patient while transitioning to clozapine therapy.	Yes
12	Mirtazapine should not exceed the maximum dose of 45 mg per day.	Depression	The patient, diagnosed with treatment-resistant depression, underwent a trial of mirtazapine at a dosage of 60 mg per day, titrated slowly to this maximum dose.	Reduced mirtazapine dosage to 45 mg per day.	No
13	It's crucial to use caution when combining linezolid and paroxetine concurrently due to the risk of interactions that could potentially lead to serotonin syndrome.	Depression	A patient diagnosed with depression and a urinary tract infection (UTI) has been initiated on a regimen consisting of linezolid 600 mg and paroxetine 20 mg.	Pauses the use of paroxetine for a week while the patient is undergoing antibiotic treatment with linezolid 600 mg.	Yes
14	The patient has exceeded the maximum daily dose of the stimulant lisdexamfetamine, which is 70 mg per day.	Autism spectrum disorder with hyperactivity	Patient was on lisdexamfetamine 80 mg per day (max is 70 mg per day)	Reduced the lisdexamfetamine dosage to 70 mg daily to adhere to the recommended maximum dose.	No
15	The use of escitalopram in a patient with ventricular tachycardia and an implantable cardioverter-defibrillator (ICD), should be approached with caution.	Depression	The patient, who suffers from depression and an implantable cardioverter-defibrillator (ICD), also presents with ventricular tachycardia and prolonged QTc interval.	Discontinue escitalopram and initiate sertraline.	Yes
16	Valproic acid should be initiated at 10–15 mg/kg/day in paediatric patients, then titrated based on age, weight, and individual response. Patient also reported adherence issues.	Bipolar disorder	For a patient with bipolar disorder weighing 75 kg, starting valproic acid at 200 mg three times daily resulted in minimal effect after one month of use.	Adjusted the dose to 1000 mg at bedtime based on the patient's current weight. Consider using the extended-release formulation of valproic acid once daily at bedtime to address adherence issues.	Yes
17	The concurrent use of St. John's Wort (Hypericum perforatum) and escitalopram, an antidepressant, may result in a potential interaction. St. John's Wort is known to influence the metabolism of certain medications, including escitalopram, by inducing liver enzymes. This can reduce the effectiveness of escitalopram and potentially diminish its therapeutic benefits. Additionally, combining multiple serotonergic agents concurrently may elevate the risk of serotonin syndrome in general.	Mild–moderate depression	During the interview, the patient disclosed using St. John's Wort in conjunction with escitalopram, highlighting a potential interaction warranting further consideration.	Advised to discontinue escitalopram	Yes
18	Fluoxetine, an antidepressant, can be excreted into breast milk, and the potential risks and benefits should be thoroughly assessed before deciding on its use during breastfeeding.	Depression	Patient with depression and lactating (newly born infant). A patient currently taking fluoxetine has been reported to be concurrently breastfeeding a new-born infant.	Discontinued fluoxetine and started sertraline	Yes
19	Class D antidepressant in pregnancy Class D is often used in pregnancy categorisation to describe medications with a potential risk of harm to the foetus. Regarding antidepressants, considered Class D includes certain selective serotonin reuptake inhibitors (SSRIs) and serotonin-norepinephrine reuptake inhibitors (SNRIs). Examples include paroxetine and venlafaxine.	Depression	The patient, who was previously being treated for depression with paroxetine, is now pregnant.	Discontinued paroxetine and switched to sertraline	Yes
20	Adjustment of CNS stimulants in paediatric patients with autism spectrum disorder and hyperactivity should be based on individual factors such as weight, age, and treatment response.	Autism spectrum disorder with hyperactivity	Patient has a diagnosis of autism spectrum disorder with ADHD-type symptoms and a lack of response to methylphenidate 5 mg twice daily. Patient's weight is 17 kg.	Adjusted medication to 7.5 mg twice per day.	No
21	Extrapyramidal side effects (EPS), such as dystonic reactions affecting the eyes, can be observed with certain long-acting antipsychotic injections. Ocular EPS symptoms may present as abnormalities in eye movements or visual disturbances attributed to the impact of antipsychotic medications on the brain's dopamine system.	Schizophrenia	The patient has reported experiencing an ocular gyres crisis, a dystonic reaction associated with the use of Paliperidone. The patient was receiving a dose of 350 mg injectable Paliperidone every 3 months.	Consider discontinuing the Paliperidone long-acting injection and transitioning to an Aripiprazole long-acting injection.	Yes
22	Insomnia and weight loss are recognised side effects associated with the use of central nervous system (CNS) stimulants, such as methylphenidate.	Attention Deficit Hyperactivity Disorder	The patient, weighing 51 kg, responded partially to Methylphenidate 18 mg taken in the afternoon.	Advised the patient to take the medication early in the morning, either before or after breakfast. The plan is to adjust the dosage to 36 mg if it is well tolerated.	Yes
23	Pancreatitis is a known side effect associated with the use of valproic acid, an anticonvulsant and mood stabiliser medication.	Schizoaffective disorder	The patient receiving valproic acid 1000 mg at bedtime has developed pancreatitis as a side effect.	Discontinued valproic acid and started carbamazepine	Yes
24	Drug–Drug interaction between lamotrigine and valproic acid. Valproic acid can increase the plasma levels of lamotrigine by inhibiting its metabolism. Combining lamotrigine with valproic acid increases the risk of developing a serious skin rash, including SJS and toxic epidermal necrolysis.	Bipolar disorder	The bipolar patient, currently on lamotrigine 150 mg twice daily, has exhibited an insufficient response, necessitating a planned transition to valproic acid.	Valproic acid can increase lamotrigine levels. It's recommended to reduce lamotrigine by 50% before starting valproic acid. Begin with a low dose of valproic acid, such as 500 mg at bedtime, and monitor valproic acid levels within 5 days.	Yes
25	In some rare instances, mirtazapine, an antidepressant, has been associated with the development of psychotic symptoms as a side effect.	Depression	The patient, being treated for depression, was initially prescribed mirtazapine at 15 mg, which was later increased to 30 mg. However, the patient has reported experiencing visual hallucinations.	The patient was advised to discontinue the medication and educated to monitor their condition closely. If symptoms persist, initiation of an antipsychotic such as quetiapine may be considered.	Yes
26	Relapse refers to the return of symptoms after a period of improvement.	Bipolar	The patient with bipolar disorder, currently on valproate 500 mg SR and clozapine 200 mg, started experiencing early signs of relapse. The patient also reported severe insomnia, which has affected their ability to work.	Suggested to add zolpidem 10 mg at bedtime and increase the clozapine dose to 300 mg at bedtime. The patient was extensively educated about relapse symptoms and instructed to visit the emergency room if symptoms worsened.	Yes
27	Antidepressant withdrawal symptoms	Depression	Patient abruptly stopped escitalopram and reported experiencing bad withdrawal symptoms, including nausea, vomiting, diarrhea, headaches, tremors, and sleep disturbances.	Education was provided regarding the importance of medication adherence, and restarted on escitalopram – a small dose of 5 mg followed by close patient monitoring.	Yes
28	Non-adherence to oral antipsychotics can be caused by several factors, including the burden of side effects, lack of social support or, complex dosing regimens, etc.	Schizophrenia	The patient diagnosed with schizophrenia is currently prescribed paliperidone 3 mg and aripiprazole 10 mg but is non-compliant with their medication regimen.	Patient was educated about the importance of medication adherence and switched to a long-acting injection of paliperidone as an additional intervention to improve compliance.	Yes

While DRPs present challenges, they can be prevented with timely identification and intervention. Addressing DRPs through identification, prevention, resolution, and proper documentation is essential to the pharmaceutical care process. Several DRP classification systems have been developed over the years. Developing a DRP classification system that is feasible, user-friendly, systematic, widely applicable in a diverse setting, and allows for enhanced evaluation and documentation can have a profound effect on resolving them effectively (Hohmann et al., 2012; Jayakumar et al., 2021; Ruths et al., 2007). In our study, the Krska DRP classification system was utilised, while other studies employed different DRP classifications such as the APS-Doc, Clinical Pharmacy Activity Classification System, Slovenian, Hepler, and Strand, among others.

The most common DRPs identified in our study were the need for patient education (16.21%), followed by inappropriate dosage regimens (13.51%), and adverse effects reported by patients from their therapy (12.35%) (Table 2). Alkhanbashi et al., in their study conducted in Saudi Arabia, identified inappropriate dosage regimens (25.6%) as one of the top three medication-related problems requiring pharmacist intervention (AlKhanbashi et al., 2024). Also, Bell et al. conducted a study on mental health patients also found that DRPs with dosage regimens (36.8%) were the most (Bell et al., 2006). This was similar to a study conducted at a Slovenian psychiatric hospital (37.8%) (Stuhec & Tement, 2021). Many significant pharmacist interventions addressing dosage issues identified in our study, as detailed in Table 3, included case numbers 4, 9, 11, 12, 14, 16, 21, and 24.

DRPs related to adverse effects in our study was found to be 12.35%. This was slightly higher than similar studies reported from other parts of Saudi Arabia (6.4% & 2.43%) (AlKhanbashi et al., 2024; Alshahrani et al., 2019). On the contrary, Jayakumar et al. and Bell et al. reported significantly higher numbers of pharmacist interventions for adverse event DRP classification among psychiatric patients, with 30.2%, and 47%, respectively (Bell et al., 2006; Jayakumar et al., 2021). Notable examples of significant adverse events related to DRPs that required pharmacist intervention, as detailed in Table 3, include cases 10, 11, and 21–23.

In another case, a patient consulted a pharmacist regarding their use of St. John’s wort while continuing escitalopram. The patient was extensively educated and advised against this combination, as St. John’s wort may enhance the serotonergic effect of escitalopram, resulting in serotonin syndrome (Badar, 2024; Benitez et al., 2022). Additionally, the pharmacist detected low thyroid levels in a patient receiving fluoxetine for depression. Subsequently, the patient was transitioned to escitalopram, and the pharmacist collaborated with the patient’s primary care provider to adjust levothyroxine doses. It is important to emphasise here that the most frequent DRPs in patients on antidepressants were primarily addressed through pharmacist intervention classified as ‘education required.’ This highlights the significant role of pharmacist-guided education in improving medication adherence in patients suffering from depression. Importantly, since the majority of DRPs necessitating pharmacist intervention involved education, it reinforces the importance of pharmacist-led patient education specifically for improving adherence to antidepressant therapy, as shown by several previous studies (Adler et al., 2004; Canales et al., 2001; Rickles et al., 2005).

In our study, a remarkably high acceptance rate (98.45%) of pharmacists interventions when compared to a study from Abha, Saudi Arabia (54.13%) (Alshahrani et al., 2019). This clearly underscores the favourable reception of clinical pharmacists in the psychiatric clinic and reinforces physicians’ confidence in the pharmacist’s role. In a comprehensive four-year prospective study within a post-surgical and post-transplantation ward, Charpiat et al. documented a 47% acceptance rate and a 19% refusal rate for pharmacist interventions (Charpiat et al., 2012). Similarly, in an assessment of the involvement of a clinical pharmacist within a general internal/gastroenterology unit, Bosma et al. revealed an 84% acceptance rate of pharmacist intervention by internal medicine specialists (Bosma et al., 2008). Stuhec et al. observed a significant difference in acceptance rates, with 88.0% (Stuhec, 2014) and 93.7% (Stuhec & Tement, 2021) in a psychiatric hospital setting in Slovenia, compared to 48.6% and 42.8% reported in a primary care medication review service (Stuhec & Gorenc, 2019; Stuhec et al., 2019) They suggested that this higher acceptance rate may be linked to increased access to a pharmacist for psychiatrists. This further supports the idea that pharmacists with broad knowledge are well-positioned to address the knowledge gap in mental health. A study at the University Hospital’s geriatric psychiatry admission unit examined the impact of a clinical pharmacist on potentially inappropriate drug prescriptions and revealed a 68% acceptance rate for pharmacist interventions (Hannou et al., 2017). The high acceptance rate, therefore, reinforces that the advantages of clinical pharmacists can be enhanced by granting them more access, such as implementing telehealth pharmacist-led psychiatric services (Arain et al., 2022). JHAH is the only hospital in Saudi Arabia where tele-psychiatric consultation is implemented. Consequently, patients’ health outcomes are improving with the results of pharmacists-led telepsychiatry consultations. It is recommended to implement various hospitals across different specialties to benefit from clinical pharmacy activities by preventing potential DRPs and managing actual DRPs.

Collaborative care between pharmacists and psychiatrists offers a promising approach to improve patient outcomes in mental health treatment. Pharmacists play a significant role in psychiatric care by providing medication management, counselling on medication adherence, and, notably, patient education (Davis et al., 2020). Moreover, comprehensive education provided by pharmacists on psychiatric medications ensures that patients comprehend their treatment regimens, potential side effects, and the importance of adherence. Their involvement is particularly crucial in mental health due to the frequent occurrence of polypharmacy in these patients. By fostering open communication during patient education, pharmacists address patients’ concerns, improving therapeutic outcomes and reducing the stigma associated with mental health treatments. Through evidence-based patient education, pharmacists significantly optimise medication efficacy and safety, ultimately enhancing the overall quality of mental health care. Several studies, including ours, highlight the importance of the pharmacist’s role as an educator. Patient education by pharmacists, as shown in previous studies, can significantly improve medication adherence, especially for patients on antidepressants, which is crucial in these patients to prevent relapse and re-admission (Adler et al., 2004; Bell et al., 2006; Bugeja et al., 2015; Canales et al., 2001; Rickles et al., 2005).

Our study results for pharmacist recommendation closely align with those reported by Richardson et al., who found that 16.6% of pharmacist recommendations involved providing educational information to the patients, including adherence aids. Interestingly, they also reported that pharmacists spent an average of 11% of their time on clinical activities related to patient education and counselling (Richardson et al., 2014). Key pharmacist educational interventions from our study, as listed in Table 3, include case numbers 17, 22, and 25–28. As mentioned in intervention case 28 of this study, medication adherence can be particularly common and challenging for psychiatric medications due to the burden of adverse effects, lack of social support, polypharmacy or complex dosing regimens. Polypharmacy is a well-established risk factor for DRPs (Jayakumar et al., 2021; Krähenbühl-Melcher et al., 2007; Garin et al., 2021). A retrospective evaluation by Hazra et al., showed a decrease in polypharmacy from 18.3 to 6.6% as a result of pharmacist-provided education (Hazra et al., 2011). Similarly, Thompson et al., in their study to outline multifaceted interventions to reduce antipsychotic polypharmacy for patients using antipsychotic medications, found that when pharmacists conducted academic detailing and facilitated a chart reminder system, there was a reduction in antipsychotic polypharmacy: OR 0.43 (0.21–0.90) (Thompson et al., 2008). It is, therefore, evident that pharmacists can play a key role in reducing the prevalence of antipsychotic polypharmacy. Interventions cases 9 and 10 from Table 3 in our study highlight instances of polypharmacy DRPs resolved by the pharmacist. Particularly, these interventions involved discontinuing the concurrent use of antidepressants from SSRI and SNRI classes to prevent the increased risk of serotonin syndrome. It is worth noting that the physicians accepted all pharmacist interventions to address DRPs through patient education.

It is important to address the limitations of our study. It is a single-center retrospective study and the sample size is relatively minimal with respect to the overall prevalence of psychiatric disorders in Saudi Arabia. Considering the substantial potential for adverse effects from psychiatric medications and the need for more stringent monitoring for assessing clinical response in patients, our study highlights the significant contribution of pharmacists in identifying and resolving DRPs.

## Conclusion

The findings of the study indicate that pharmacists with expertise in psychiatric pharmacotherapy make a valuable contribution to the care of patients with mental illness. This is demonstrated by improved patient access to pharmacists through the JHAH telehealth mental clinic, where pharmacists identify drug-related problems and recommend various therapeutic interventions promptly to resolve them. These interventions primarily involve patient education, dose adjustments, and the identification of potential adverse drug reactions, all aimed at enhancing medication safety. Additionally, these pharmacist-led interventions were widely accepted by psychiatric physicians, as demonstrated by the high acceptance rate of the recommendations. The study emphasised the crucial role of pharmacists in managing DRPs for patients on psychotropic medications and the importance of ensuring these medications are used safely and effectively.
